# *In vitro* and ex vivo immunomodulatory effects of human placental mesenchymal stem cells in hidradenitis suppurativa

**DOI:** 10.3389/fimmu.2025.1642014

**Published:** 2025-09-25

**Authors:** Vaiva Jariene, Paulius Valiukevicius, Ruta Insodaite, Ugne Janonyte, Romaldas Maciulaitis, Justinas Maciulaitis, Astra Vitkauskiene, Evelina Zemaite, Christos C. Zouboulis, Skaidra Valiukeviciene

**Affiliations:** ^1^ Department of Skin and Venereal Diseases, Lithuanian University of Health Sciences, Kaunas, Lithuania; ^2^ Department of Skin and Venereal Diseases, Hospital of Lithuanian University of Health Sciences Kauno Klinikos, European Reference Network for Rare and Complex Diseases of the Skin (ERN Skin) member, Kaunas, Lithuania; ^3^ Department of Neurology, Lithuanian University of Health Sciences, Kaunas, Lithuania; ^4^ Department of Neurology, Hospital of Lithuanian University of Health Sciences Kauno Klinikos, Kaunas, Lithuania; ^5^ Institute of Physiology and Pharmacology, Lithuanian University of Health Sciences, Kaunas, Lithuania; ^6^ Institute of Biology Systems and Genetic Research, Lithuanian University of Health Sciences, Kaunas, Lithuania; ^7^ Institute of Cardiology, Lithuanian University of Health Sciences, Kaunas, Lithuania; ^8^ Department of Laboratory Medicine, Lithuanian University of Health Sciences, Kaunas, Lithuania; ^9^ Departments of Dermatology, Venereology, Allergology and Immunology, Staedtisches Klinikum Dessau, European Reference Network for Rare and Complex Diseases of the Skin (ERN-Skin) member, Brandenburg Medical School Theodor Fontane and Faculty of Health Sciences Brandenburg, Dessau, Germany

**Keywords:** hidradenitis suppurativa, acne inversa, mesenchymal stem cells (MSC), treatment, hidradenitis suppurativa-acne inversa-therapy

## Abstract

**Background:**

Hidradenitis suppurativa is a chronic inflammatory skin disease marked by immune dysregulation and elevated pro-inflammatory cytokines. While biologics like adalimumab target specific pathways, their limited efficacy highlights the need for broader immunomodulatory treatments. Mesenchymal stem/stromal cells (MSCs) have shown promise due to their immunosuppressive properties and ability to modulate both innate and adaptive immunity. This study investigates the effects of naïve (n-MSCs) and cytokine-preactivated (a-MSCs) placental MSCs on the immune responses in HS.

**Methods:**

MSCs were isolated from healthy term placentas and either used naïvely or preactivated with IFN-γ and TNF-α. Peripheral blood mononuclear cells (PBMCs) from HS patients (n=3) and healthy donors (n=3) were co-cultured with n-MSCs, a-MSCs, or adalimumab. Additionally, lesional, perilesional, and healthy 4 mm in diameter skin punch biopsies from 10 HS patients and 3 controls were cultured in a transwell system with the same interventions. Flow cytometry assessed lymphocytes proliferation and T cell subsets while Luminex assays measured cytokine levels.

**Results:**

Both n-MSCs and a-MSCs significantly inhibited lymphocytes proliferation and shifted T cell populations, increasing CD4+ and decreasing CD8+ T cells. The a-MSCs notably reduced IL-17A and IFN-γ in PBMC co-cultures; n-MSCs had partial effects. HS skin explants exhibited elevated IL-1β, IL-10, and IL-17A compared to healthy skin. The n-MSCs markedly reduced all three cytokines in lesional and perilesional tissues. Moreover, a-MSCs selectively increased IL-10 in lesional skin.

**Conclusion:**

Placental MSCs, especially in their naïve form, demonstrate potent immunomodulatory effects by reducing pro-inflammatory cytokines and altering T cell dynamics in HS. Compared to adalimumab, MSCs offer a broader immunoregulatory profile, suggesting their potential as a multitarget therapy for HS. These findings support further clinical investigation of MSC-based treatments in managing this complex disease.

## 1 Introduction

Hidradenitis suppurativa/acne inversa (HS) is a potentially severe, chronic, inflammatory, recurrent skin disease of the hair follicle. Some of its variants belong to the rare diseases (Orphanet codes 387, 289478) (1). A meta-analysis by Jfri et al. estimated the prevalence of HS at 0.40% (2). Although the precise pathogenesis of HS remains uncertain, current perspectives are shifting from the traditional view of HS as primarily a disorder of follicular occlusion to a model emphasizing autoinflammatory keratinization (3). Evidence indicates that HS may involve inflammatory, autoimmune, or a combination of both mechanisms, with hyperkeratotic plugging of the follicular infundibulum identified as a central pathogenic event (4). Clear evidence suggests the involvement of proinflammatory cytokines in the immune dysregulation of HS (4). Notably, the interleukin-1 (IL-1) family of cytokines (IL-1α and IL-1β) (5) as well as multiple IL-36 transcripts (6) are elevated in HS lesions as demonstrated by ribonucleic acid (RNA) sequencing and real-time reverse transcription polymerase chain reaction (qRT-PCR). IL-23A (encoding IL-23p19) transcripts are elevated in HS-affected tissues, and expression of IL-23A mRNA has been found in activated macrophages in HS lesions using qRT-PCR and IL-23 subunits by immunohistochemistry and immunofluorescence confocal microscopy (7). Multiple IL-17 family transcripts are elevated in HS lesions, including IL-17A and the resultant protein IL-17A as well as IL-17C and IL-17F, the latter forming heterodimers with IL-17A (8).

The most favorable anti-inflammatory treatment for HS is biologics that directly target cytokines. At present, the only European Medicines Agency (EMA) and US Food and Drug Administration approved medications for the treatment of HS are the tumor necrosis factor α (TNF-α) inhibitor adalimumab, the IL-17A inhibitor secukinumab, and the IL-17A/F inhibitor bimekizumab (9). Preclinical *in vivo* or *ex vivo* studies were published for adalimumab and bimekizumab (10, 11). In general, the optimal target for successful HS treatment remains undiscovered.

Previous studies have suggested that mesenchymal stem/stromal cells (MSCs) possess intrinsic immunosuppressive capabilities that can alleviate inflammation and immune responses (12). MSCs obtained from different tissues inhibit the production of IFN-γ, TNF-α, and IL-1β (13, 14) and promote the synthesis of TGF-β and IL-10 cytokines (13). They can also suppress inflammation by means of cell-to-cell contact, extracellular vesicles, and various other secreted factors such as IDO, iNOS, HGF, TSG6, and CCL2 (15). These findings suggest MSCs as a potential multitarget treatment approach for HS, addressing both innate and adaptive inflammation.

The safety of envisioned cell therapy methods was proven at both preclinical as well as clinical phases, concluding safe clinical administration of allogeneic MSCs (16). The low expression of class I and II human leukocyte antigen (HLA) antibodies on MSCs reduces the risk of rejection in allogeneic applications in normal immunocompetent hosts, making them safe for universal application and cryopreserved, off-the-shelf product storage (17). This approach streamlines treatment processes and enhances accessibility for critical interventions.

In our previous pilot study, we investigated the immunomodulatory effects of MSCs on peripheral blood mononuclear cells (PBMCs) from 3 healthy controls and 3 HS patients. Our results showed that MSCs can effectively suppress PBMC proliferation and inhibit the production of inflammatory cytokines. Furthermore, pre-activation of MSCs with IFN-γ and TNF-α before use may enhance their therapeutic efficacy (18).

In the current study, we developed an experiment using *in vitro* PBMCs and *ex vivo* HS explant models to comprehensively evaluate the therapeutic potential of MSCs and alleviate the systemic (PBMCs) and localized (explant) immune aberrant responses that are pathogenic in HS. To our knowledge, no prior study investigating MSCs’ immunomodulatory effects on both PBMCs *in vitro* and HS explants *ex vivo* has been published before.

## 2 Materials and methods

### 2.1 Development of MSCs in 2D multilayer vials and their characterization

MSC culture and activation: To obtain MSCs, placenta was collected from healthy volunteers who underwent caesarean section after providing written informed consent. The umbilical cord, amniotic and chorionic layers were discarded, and the remaining placenta was minced and treated with 0.1% collagenase (NB6, Nordmark) and DNase solution (Pierce Nuclease, Thermo Fisher) to isolate the cells. The cell suspension was washed and filtered before being resuspended in DMEM containing 10% fetal bovine serum (Gibco) and plated in a tissue culture flask (Cell Factories, Corning) for incubation at 37°C in a humidified atmosphere containing 5% CO_2_. For activation of MSCs (a-MSCs), the fifth passage cell medium was replaced with IFN-γ (20 ng/ml; Sigma-Aldrich) and TNF-α (20 ng/ml; Sigma-Aldrich), and the cells were incubated for 24 h. The conditioned cell medium was then centrifuged at 10,000 × g for 15 min to remove cell debris, and the resulting supernatant was stored at -80°C for later use.

### 2.2 Study population and sample collection

Ten HS patients at Hurley stage II were included in the study. All patients had not been treated with systemic antibiotics for at least 6 weeks prior to enrolment. They were also biologically naïve and did not have any other inflammatory disease. Patients exhibited at least moderate disease severity according to the IHS4 score. The clinical data indicate that our research cohort is representative of moderate or severe HS severity and relatively homogeneous [mean IHS4 score 8.5 (SD 2.2)] (**Table 1**). Four mm in diameter skin punch biopsies were taken from axillary HS lesional (inflammatory nodules) and HS perilesional (at a distance of ≥5 cm from visible HS inflammation area) skin areas (n=10). Three healthy control subjects (3 females, mean age 25 years, range 23–29 years) were included in the study, and 4 mm in diameter skin punch biopsies were taken from axillary healthy skin. Finally, the collection of blood samples from HS (n=3) and healthy (n=3) study subjects was conducted under aseptic conditions. Approval by the regional bio-ethics committee was obtained (Nr. BE-2-105), and each patient and control subject gave written informed consent.

**Table 1:** Demographic and clinical characteristics of patients with hidradenitis suppurativa and healthy controls.

Patient group	HS patients biopsy group	HS patients PBMC group	Healthy patients
Male Female ratio	8:2	3:0	0:3
Mean age (SD)	43.6 (10.1)	37 (2)	25 (3.5)
Mean disease duration (SD)	10.7 (7.4)	8.7 (3.8)	–
Mean IHS4 Score (SD)	8.5 (2.2)	9 (3)	–
Mean BMI (SD)	35.1 (7.5)	33.2 (2.2)	20.6 (1.5)
Positive hypertension anamnesis (Yes/No)	4/6	1/2	0/3
Positive diabetes anamnesis (Yes/No)	2/8	0/3	0/3
Positive Smoking Anamnesis(Yes/No)	8/2	3/0	0/3
Mean CRP level (SD)	7.2 (3.8)	10.3 (5.2)	5 (0)

SD, standard deviation; BMI, body mass index; IHS4, International Hidradenitis Suppurativa Severity Score System; CRP, C-reactive protein; PBMC, peripheral blood mononuclear cells.

### 2.3 Flow cytometry

PBMCs were isolated from HS patients and healthy volunteers using the method described by Oliver Vila et al. in 2018 (19). The isolation process involved gradient centrifugation to separate PBMC from venous blood, followed by staining with CFSE and stimulation with PHA. PBMCs were cultured in RPMI 1640 cell culture medium (Gibco) with 10% FBS. Additionally, patient PBMCs were co-cultured with either naive mesenchymal stem cells (n-MSCs), activated mesenchymal stem cells (a-MSCs), or high-dose 30 μg/ml adalimumab (HUMIRA^®^, AbbVie) to create the following groups: 1) n-MSCs, 2) a-MSCs, 3) adalimumab, 4) HS control and 5) healthy control. Concentrations of adalimumab were chosen according to previously published studies (10), and maximum concentrations were specified in the summary of product characteristics.

PBMCs were collected after 5 days and labeled with a panel of antibodies and stains: 7-AAD for viability, anti-CD3, anti-CD4, anti-CD8, anti-CD25 and anti-CD127. They were then subjected to flow cytometry analysis, which involved examination of 100,000 events per sample. The data obtained from the flow cytometry analysis were analyzed using FlowJo version 10.8.1 (BD, Ashland, Oregon, USA). The following populations were studied: live cells, CD3+ (lymphocytes), CD4+CD8- (T lymphocytes), CD4-CD8+ (Cytotoxic lymphocytes), CD4+CD25hiCD127lo (Treg lymphocytes). Overall proliferation of live cells as well as proliferation and relative counts of the above mentioned cell populations were studied. Normalized proliferation was calculated as described by Vila et al. (19). Briefly, absolute proliferation was calculated by subtracting the background proliferation in the non-stimulated condition from the proliferation in the stimulated condition. To assess the effect of coculture on proliferation, coculture absolute proliferation values were normalized to the absolute proliferation observed in stimulated single PBMC cultures.

### 2.4 Co-culture experiment

Skin biopsy and MSC co-cultures were performed following the method described by Vossen et al. (20) with minor modifications. Biopsies were placed immediately after the procedure in punched-out 3 mm holes in the Transwell membrane (0.4 μm, cellQART, SABEU, Northeim, Germany) of a 12-well plate with the epidermis exposed to the air and the dermis immersed in 1 ml of DMEM (Sigma-Aldrich, St. Louis, MO) containing 0.5% heat-inactivated human AB serum (Sigma-Aldrich) and 0.1% of gentamycin (Gibco, Waltham, MA). 200,000 a-MSCs, n-MSCs (5.26x104 cells/cm^2^), or adalimumab (30 μg/ml) were added to the respective well resulting the following groups: 1) culture as negative control; 2) lesional control; 3) lesional a-MSCs; 4) lesional n-MSCs; 5) lesional adalimumab; 6) perilesional control; 7) perilesional a-MSCs; 8) perilesional n-MSCs; 9) perilesional adalimumab, 10) healthy individual control. Skin biopsies were incubated for 24 h at 37°C in an atmosphere of 5% CO_2_ and 98% humidity. Supernatants were centrifuged at 10,000 g for 1 min to remove cell debris and then stored at −80°C for later analysis.

### 2.5 Cytokine Luminex analysis

Cytokine protein concentrations in the supernatant were assessed using a custom-designed premixed Luminex Discovery assay (Bio-Techne, MN, USA) for the following cytokines: IL-1β, IL-10, IL-17, IFN-γ and TNF-α. Values extrapolated from the standard curves were considered unreliable. Thus, a concentration = 0 pg/ml was assigned.

### 2.6 Statistical analysis

The results are presented as median values along with the interquartile range (minimum to maximum values). Statistical analysis was conducted using GraphPad Prism version 10.0.0 (GraphPad Software), considering a significant level of p<0.05. Group differences were assessed using the Kruskal-Wallis test, while Dunn’s test was applied for pairwise comparisons.

## 3 Results

### 3.1 Flow cytometry

**Figure 1:**
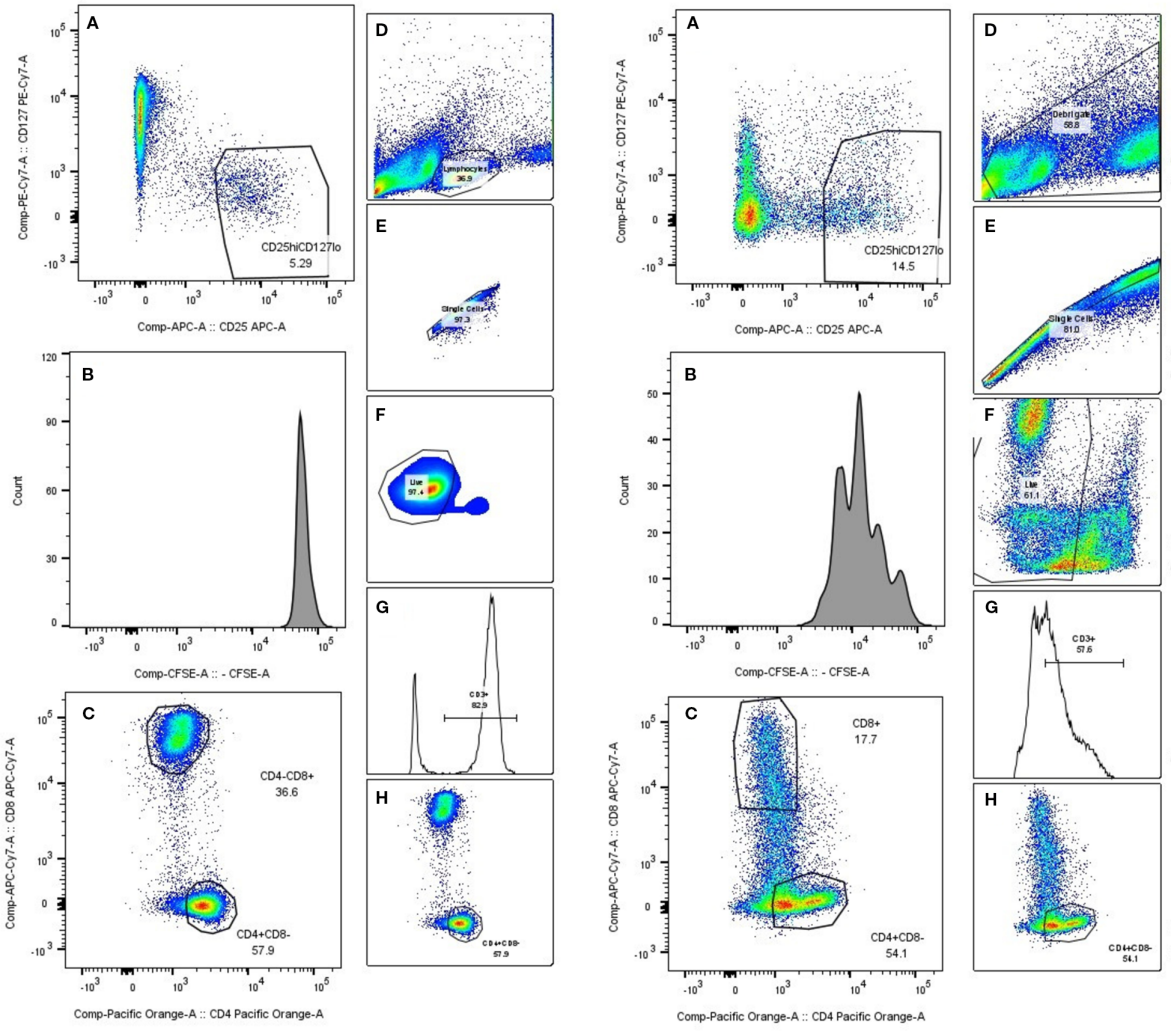
PBMC gating. A representative gating schematic. On the left side collage of PHA unstimulated PBMCs. Right side stimulated PBMCs. **(A)** Treg selection, **(B)** CFSE histogram, **(C, H)** CD4/CD8 selection, **(D)** lymphocyte population selection based on size and scatter, **(E)** singlet gate, **(F)** live cell gate, **(G)** CD3 gate.

**Figure 2:**
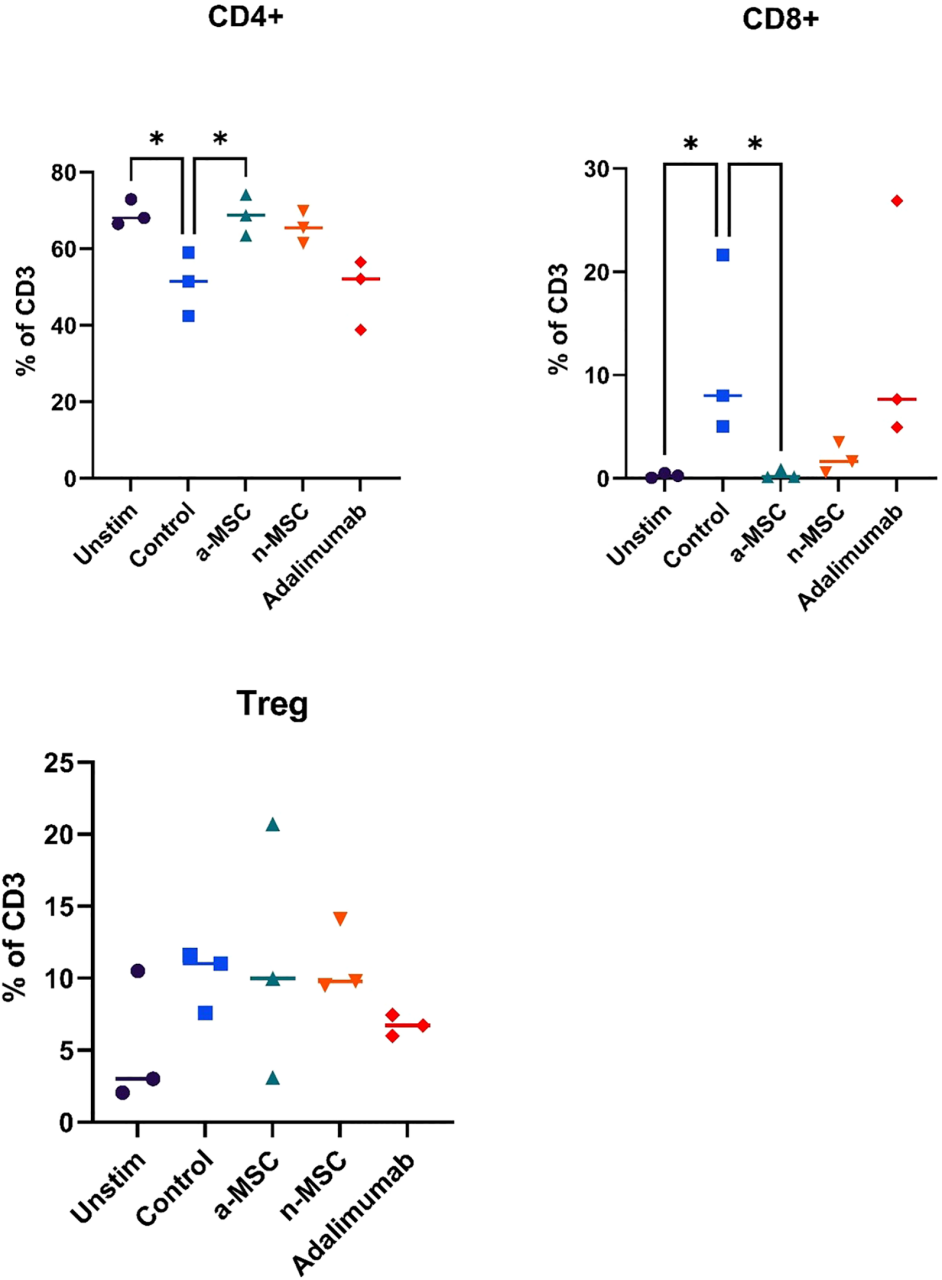
Lymphocyte subtype proliferation. n = 3, single technical replicate. a-MSCs, activated mesenchymal stem cells; n-MSCs, naïve mesenchymal stem cells; Unstim, PHA unstimulated PBMCs; Control, PHA stimulated PBMCs. Group differences were assessed using the Kruskal-Wallis test, followed by Dunn’s *post hoc* test for pairwise comparisons. *p < 0.05.

Stimulation of PBMCs with PHA resulted in robust lymphocyte proliferation, as seen in **Figure 1B**, while comparing unstimulated (left) and stimulated (right) samples, changes in the CFSE histogram show dilution of CFSE, resulting from cell division. Four peaks are seen in the stimulated sample, corresponding to four generations of cells. We compared lymphocyte proliferation and relative changes in CD4, CD8 and Treg populations across different interventions (**Figures 1A, C, D–H**). CFSE dye was used, which is diluted as the cell proliferates. Both n-MSCs and a-MSCs inhibited overall lymphocytes proliferation (**Figure 2**, CD3 proliferation), as well as CD4 and Treg lymphocyte proliferation. Tregs n-MSCs resulted in a tendency for reduction, which did not reach significance. There were no significant differences in CD8 lymphocyte proliferation. Adalimumab did not show a significant effect on the overall lymphocyte proliferation.

**Figure 3:**
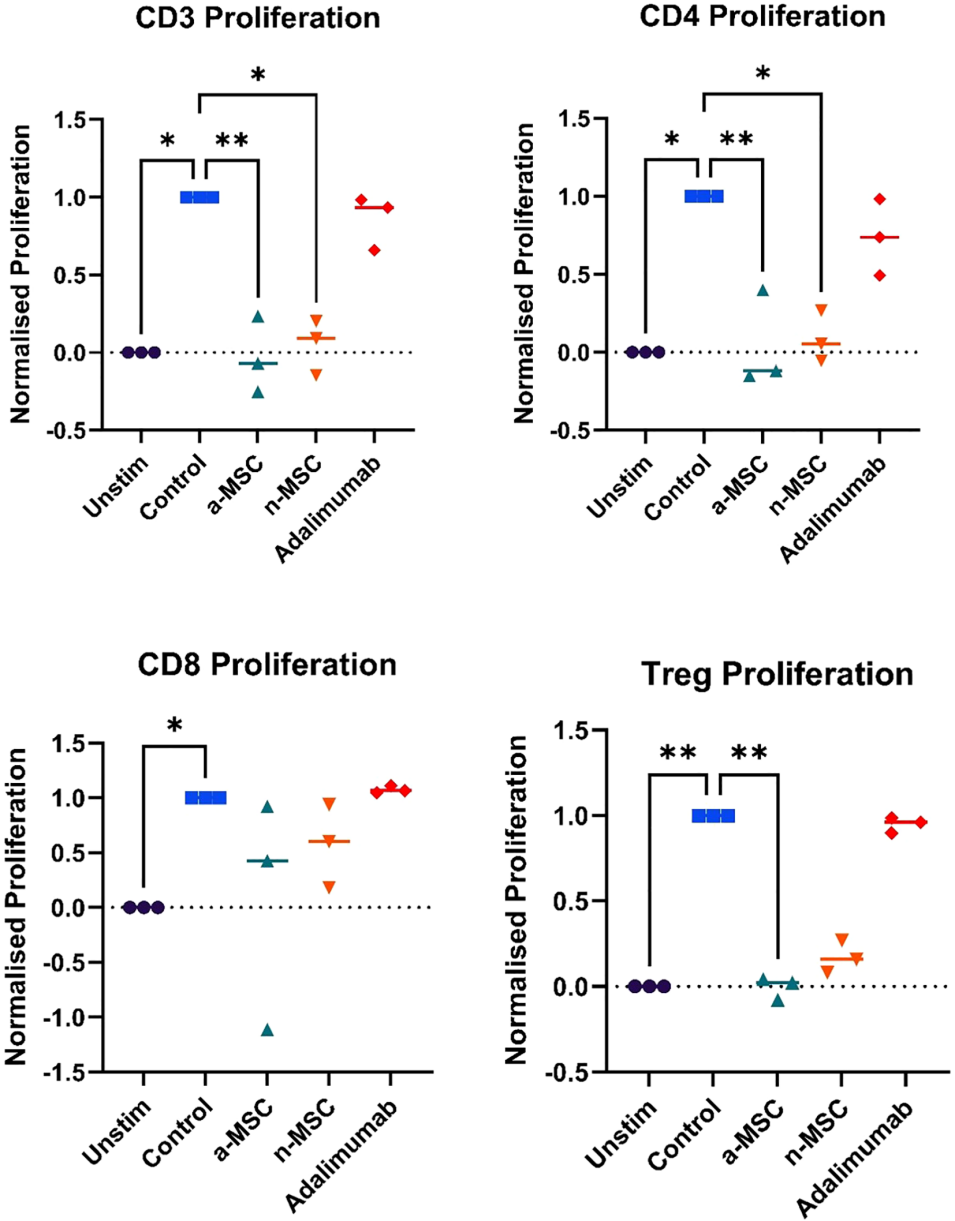
Changes in lymphocytes relative counts. n = 3, single technical replicate a-MSCs, activated mesenchymal stem cells; n-MSCs, naïve mesenchymal stem cells; Unstim, PHA unstimulated PBMCs; Control, PHA stimulated PBMCs. Group differences were assessed using the Kruskal-Wallis test, followed by Dunn’s *post hoc* test for pairwise comparisons. *p < 0.05, **p<0.01.

Interestingly, when analyzing changes in CD4, CD8 or Treg distribution across different interventions, several significant results stood out (**Figure 3**). While both a-MSCs and n-MSCs reduced the proliferation of CD4 lymphocytes, neither intervention reduced the relative count. In fact, in the samples co-cultured with a-MSCs, we found a significant increase in CD4 lymphocyte percentage. Additionally, the amount of CD8 lymphocytes was greatly reduced in the overall lymphocyte population. As for Tregs, there were no significant differences across interventions.

Overall, these results show that a-MSCs and n-MSCs not only reduced lymphocyte proliferation but induced a shift in distribution, resulting in a greatly decreased amount of CD8 (cytotoxic lymphocytes), and an increase in CD4 (helper lymphocytes). While both a-MSCs and n-MSCs reduced Treg proliferation, this did not result in a reduced percentage of regulatory lymphocytes in the population, which are important for reducing inflammation. Therefore, in the samples with a-MSCs and n-MSCs, we can see a shift in balance towards a less cytotoxic lymphocyte population.

### 3.2 Cytokine Luminex analysis

**Figure 4:**
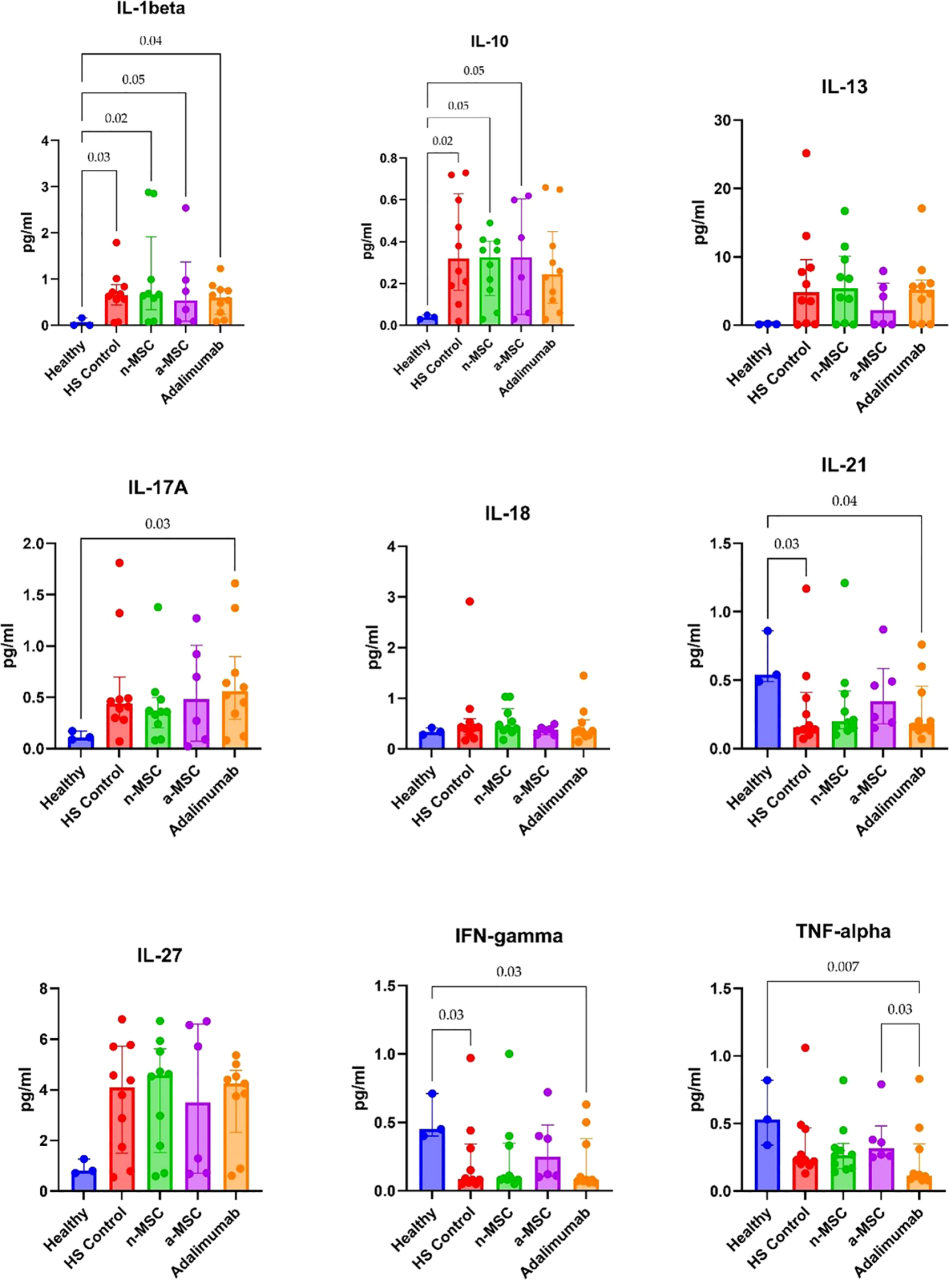
Cytokine concentrations in PHA stimulated PBMC samples. n = 3, measured in duplicates a-MSCs, activated mesenchymal stem cells; n-MSCs, naïve mesenchymal stem cells; HS, hidradenitis suppurativa; IFN, interferon; IL, interleukin; TNF, tumor necrosis factor. Group differences were assessed using the Kruskal-Wallis test, followed by Dunn’s *post hoc* test for pairwise comparisons. Significant p-values (< 0.05) are indicated.

Cytokine concentration in PBMC supernatants from HS patients and healthy individuals was analyzed. HS patient PBMCs were subjected either to regular cell culture medium (HS control) or co-cultured with n-MSCs, a-MSCs or adalimumab. They produced greater amounts of cytokines after stimulation (**Figure 4**), which shows an aberrant systemic immune response. When comparing cytokine concentrations across different interventions, we found that a-MSCs statistically significantly reduced pro-inflammatory cytokines (IL-17A, IFN-γ) and anti-inflammatory IL-10 levels (**Figure 4**) when compared with HS control. Naive MSCs showed partial effects.

**Figure 5:**
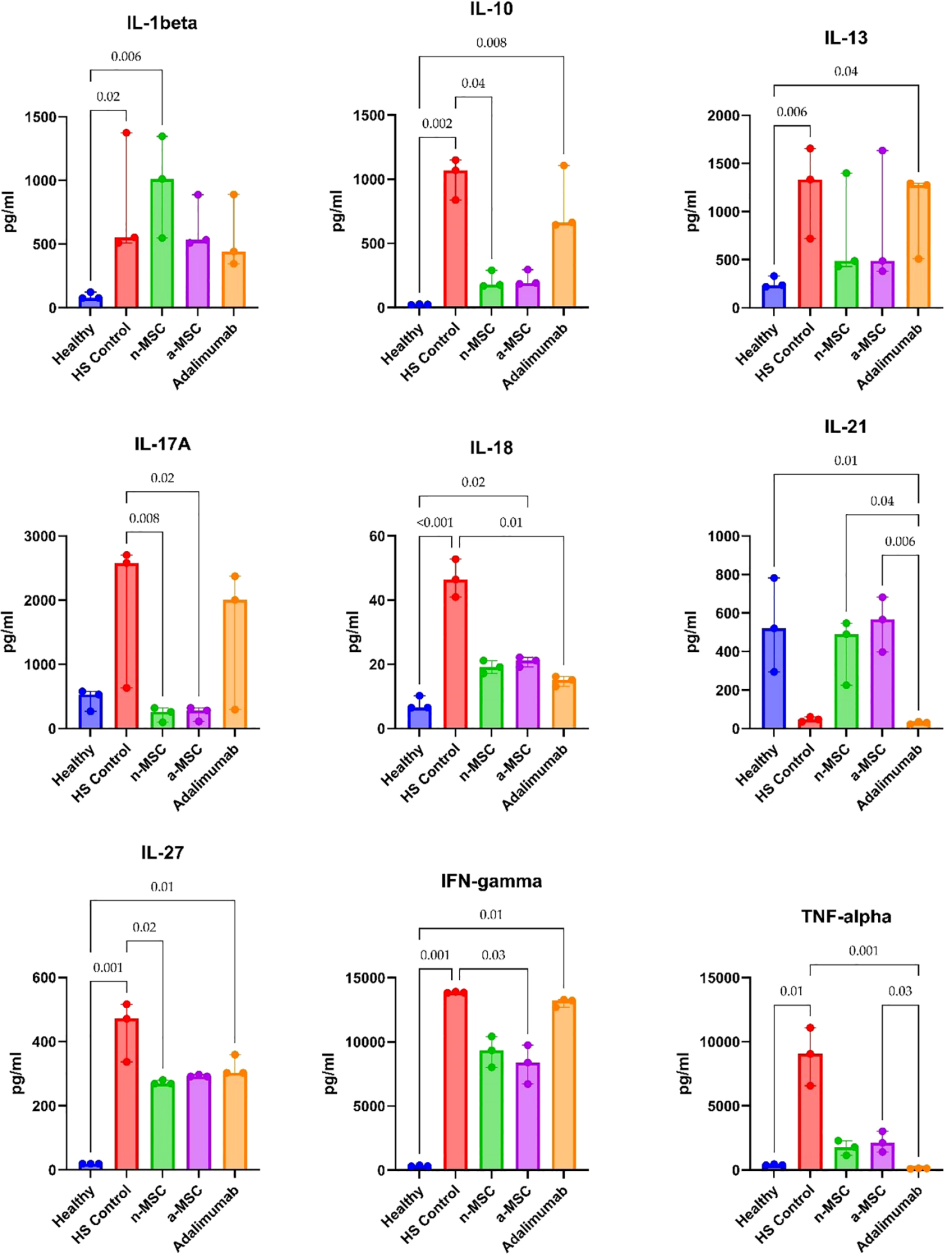
Cytokine concentrations in lesional skin biopsy samples. n = 3 for healthy subjects, n = 10 for adalimumab. a-MSCs, activated mesenchymal stem cells (n = 7); n-MSCs, naïve mesenchymal stem cells (n = 10); HS, hidradenitis suppurativa (n = 10); IFN, interferon; IL, interleukin; TNF, tumor necrosis factor. Measured in duplicates. Group differences were assessed using the Kruskal-Wallis test, followed by Dunn’s *post hoc* test for pairwise comparisons. Significant p-values (< 0.05) are indicated.

**Figure 6:**
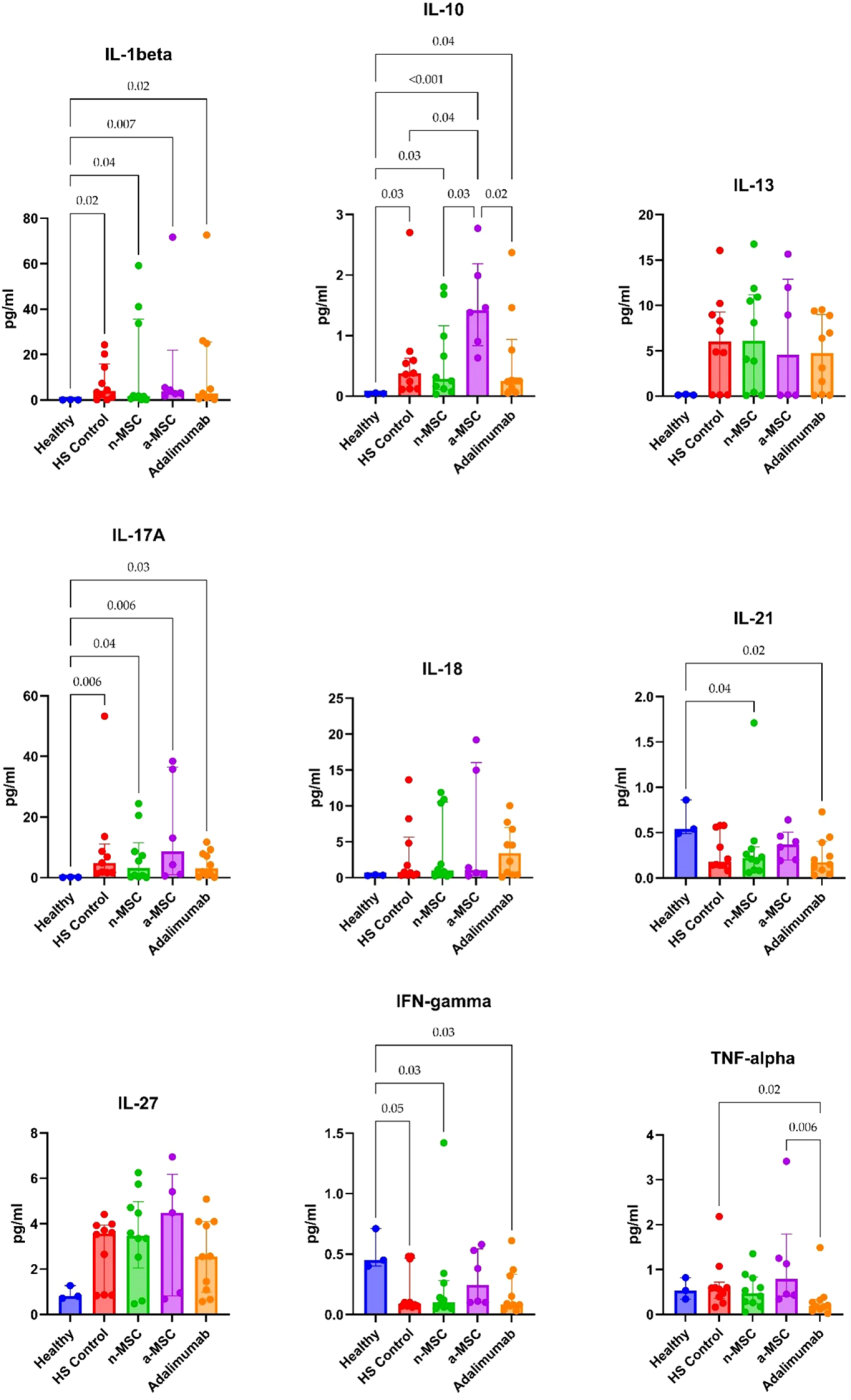
Cytokine concentrations in perilesional skin biopsy samples. n = 3 for healthy subjects, n = 10 for adalimumab. a-MSCs, activated mesenchymal stem cells (n = 7); n-MSCs, naïve mesenchymal stem cells (n = 10); HS, hidradenitis suppurativa (n = 10); IFN, interferon; IL, interleukin; TNF, tumor necrosis factor. Measured in duplicates. Group differences were assessed using the Kruskal-Wallis test, followed by Dunn’s *post hoc* test for pairwise comparisons. Significant p-values (< 0.05) are indicated.

For the *ex vivo* model, we examined lesional and perilesional biopsies from HS patients and healthy individuals. HS patient biopsies were grown in regular medium (HS control) or co-cultured with n-MSCs, a-MSCs or adalimumab. The lesional control biopsies from HS patients showed significantly elevated IL1-β, IL-10 and IL-17A concentrations when compared with healthy controls (**Figure 5**). In perilesional biopsy samples, HS control patients’ results showed significantly elevated IL1-β and IL-10 concentrations when compared with healthy controls (**Figure 6**).

In lesional biopsies, n-MSCs showed a tendency to reduce inflammatory cytokines (IL-1β, IL-17A, TNF-α) and IL-10 concentrations when compared with the HS control group. Interestingly, a-MSCs statistically significantly increased IL-10 concentration when compared with HS control samples (**Figure 5**).

In perilesional biopsies, n-MSCs demonstrated reductions in pro-inflammatory cytokines (IL-1β, IL-17A, TNF-α) and stabilized IL-10 levels (**Figure 6**). Overall, these results show that the local immune response in HS patient skin is pro-inflammatory. Robust reduction of inflammation was seen in n-MSC samples, followed by a lesser reduction in a-MSC samples. Adalimumab also reduced inflammation, but it affected a smaller number of pro-inflammatory cytokines.

## 4 Discussion

Previous studies have established that MSCs possess the ability to prevent immune system overstimulation through the modulation of various inflammatory pathways (12). Although the mechanisms of MSC-mediated regulation on inflammation are clear, MSCs are reported to suppress T cell proliferation and differentiation, as well as inhibit Th1 and Th17 activity, and promote Treg induction and IL-10 production (21). Thus, MSCs are prone to sense multifactorial signals and modulate various proinflammatory cytokines, such as TNF-α, IL-1β and IFN-γ (22, 23). Two clinical studies have demonstrated that intralesional injection of allogeneic MSCs in combination with adalimumab-maintained therapy (24) or surgery (25) can induce clinical remission of persistent HS fistulas. Their authors hypothesized that intralesional MSCs may have regulatory effects on inflammatory mediators, including Th1 and Th17 cells, macrophages, and dendritic cells, as well as their regenerative properties that promote tissue healing (24). Other studies highlighted the immunomodulatory potential of adipose tissue-derived MSCs (26, 27), suggesting their ability to upregulate IL-10 secretion by dendritic cells, leading to a less potent immune response or even T cell anergy (26). Interestingly, MSCs derived from patients with skin inflammatory diseases often demonstrate compromised inflammation-regulating abilities (28). For example, MSCs derived from HS patients are characterized by elevated levels of several helper T-cell cytokines (IL-6, IL-10, IL-12, IL-17A, IFN-γ) (29), leading to the hypothesis that MSCs are involved in HS pathogenesis at certain inflammation stages.

In our study, n-MSCs showed a consistent trend of reducing IL-1β, IL-17A, and TNF-α in both lesional and perilesional HS samples, while a-MSCs significantly decreased IL-17A and IFN-γ in PBMC samples. Although adalimumab also reduced inflammation, it had a more limited effect, targeting fewer pro-inflammatory cytokines (10). Our experimental results suggest that MSCs may have broader immunomodulatory potential for the treatment of HS compared to adalimumab. Another key observation is that MSCs inhibited the overall lymphocyte proliferation and reduced the number of CD8 lymphocytes, shifting the population towards a less cytotoxic profile. For instance, research has demonstrated that MSCs are able to downregulate CD8 expression on CD8 cells, therefore promoting a less aggressive immune response (30), which could be beneficial in such conditions as HS, as it is characterized by excessive autoinflammation.

It is worth mentioning that the HS cytokine pathway is also associated with the anti-inflammatory mediator IL-10, which suppresses T-lymphocyte activation and inflammatory cytokine production (31, 32). The elevated levels of IL-10 may indicate T-reg cell recruitment to reduce inflammation as a response to early macrophage activation (33). Consistent with our findings, IL-10 has been found to have a marked expression in the lesional and perilesional skin of HS patients (34, 35). Other anti-inflammatory cytokines in HS, such as IL-4 and IL-13, inhibit IL-1β synthesis while stimulating IL-1 receptor antagonist production (32). Moreover, IL-13 is reported to correlate inversely with the presence of Th1/Th17-associated cytokines (36). Based on our findings, IL-10 levels were significantly elevated in lesional/perilesional skin and PBMC samples of HS patients when compared to healthy controls. However, the effect of MSCs on IL-10 expression proved to be inconsistent. In the PBMC model, MSCs reduced IL-10 levels, while the skin biopsy co-culture model with MSCs had higher IL-10 concentrations. We hypothesize that this could be explained by inherent differences between the models. In the PBMC co-culture model, MSCs reduced lymphocyte proliferation, leading to a smaller overall lymphocyte count in each culture well, which reduced the overall cytokine production. On the other hand, in the skin biopsy model, during the 24-h MSC co-culture period, the cell count is expected to remain stable, as the effect on cytokine production is likely due to changes in transcription and translation. Interestingly, this discrepancy also reflects the inherent biological differences between systemic and local immune environments. The IL-10 is secreted by a wide range of immune and non-immune cells, including regulatory T cells, monocytes, dendritic cells, macrophages, and keratinocytes. The abundance, activation status, and microenvironmental context of these cells differ significantly between peripheral blood and chronically inflamed skin. In HS, the local skin environment is characterized by sustained inflammation and tissue stress, which may induce IL-10 as a compensatory, though often insufficient, anti-inflammatory response.

Moreover, we determined significantly elevated IL-1β and IL-17A cytokines in HS lesional skin when compared with healthy controls, with a marked increase in perilesional skin and PBMC samples as well. IL-17 is known to promote neutrophil activation and upregulate the expression of proinflammatory molecules (e.g. S100A7, S100A8, S100A9) (31). Data from several previous studies report an upregulation of this cytokine in HS-affected skin (10, 34, 37–39). Moreover, HS-affected skin is characterized by a Th17-skewed cytokine profile, with cells expressing CD161 and IL-17, thus creating a dysregulation of the Th17: Treg cell axis (40). IL-17 levels are not only found to be elevated in HS patients’ serum but also tend to be higher with more advanced disease stages (41).

The methodological choices in this study were carefully made to ensure experimental reliability, biological relevance, and reproducibility. While we acknowledge that *in vivo* experiments could offer additional insights, our primary aim was to evaluate the immunomodulatory potential of MSCs using well-established, human-based models. To date, only one *in vivo* model of HS has been published, which is limited in scope and does not adequately reflect the complexity of the human disease (42). Moreover, in adherence to the 3R principles and considering the interspecies challenges associated with using human-derived MSCs in animal models, we chose to rely on validated *in vitro* and ex vivo systems. As shown in our previous studies (18), the PBMC model employed in this study has consistently provided reliable and interpretable results. We also utilized an ex vivo skin explant culture system that closely reflects the local immune microenvironment in HS lesions. Our decision to use allogeneic, placenta-derived MSCs was based on the results of previous studies (17). They demonstrated several superior characteristics of MSCs - primarily in terms of manufacturing feasibility and therapeutic potency. Placenta-derived MSCs were obtained from placental tissue, a readily available and ethically acceptable source following caesarean section. These MSCs are known to possess superior proliferative capacity compared to MSCs from other sources, likely due to their early life origin (43), making them particularly suitable for future clinical applications. For MSC activation, we selected IFN-γ and TNF-α based on robust evidence supporting their ability to prime MSCs (44). Although stimulating MSCs with patient-derived serum or lesion fluid might provide a more disease-specific inflammatory milieu, such an approach would introduce significant variability and potential unwanted effects, also due to immunological incompatibility. In contrast, the administration of defined concentrations of IFN-γ and TNF-α ensured standardized and reproducible activation. In future studies, we plan to integrate more advanced human skin models, such as the 3D-SeboSkin (10), to enhance the translational relevance and complexity of our experimental platform.

The limitations of this study include several aspects. Firstly, the size of the samples was small, which may limit the generalizability of the findings to the entire HS population. HS is a clinically heterogeneous disease. We chose a focused approach in order to detect at least minimal effects in any HS subpopulation. Consequently, we successfully included a relatively small HS patient cohort by employing strict inclusion criteria (e.g., biologic-naïve patients, no antibiotic treatment for at least six weeks). The statistically compelling results we obtained allowed us to draw conclusions regarding preliminary efficacy in the selected population. These results cannot be generalized to the entire HS population but should be viewed as a starting point for further confirmatory studies, both in the same patient group and in other HS subpopulations. Future studies are needed to include a broader representation of the HS population, with a larger number of patients and controls. Secondly, only adalimumab was used as a positive control, meaning that no direct comparison was made with the gold standard antibiotics. At this stage of MSCs development we did not intend to compare all potential alternatives including other biologics (secukinumab and bimekizumab), that target additional inflammatory axes relevant to HS pathogenesis (e.g., IL-17A and IL-17A/F). The absence of these comparators in this study is a limitation, and future investigations will aim to include these agents. Additionally, there is a growing body of evidence supporting the use of more advanced HS skin models, such as the 3D-SeboSkin model (10), which could be utilized for the *ex vivo* part of the study. This study provides a preliminary but important assessment of the immunomodulatory effects of MSCs in HS. The results demonstrate consistent changes in key cytokines such as IFN-γ and IL-17A and reveal novel findings on compartment-specific modulation of IL-10. Together, these observations offer insight into potential immunological targets of MSCs and lay a critical foundation for future in-depth mechanistic investigations.

To conclude, our findings further confirm that HS is driven by complex proinflammatory cytokine pathway dysregulation, as stated in previous studies of HS immunopathogenesis. Accordingly, MSC-based therapies should come into consideration and be deemed as a promising therapeutic intervention on account of their ability to modulate several immune pathways, thereby decreasing inflammation driven by HS. Our approach in applying *in vitro* PBMCs and *ex vivo* HS explant models allow for evaluation of MSCs’ therapeutic potential in alleviating systemic (PBMCs) and localized (explant) immune aberrant responses, which are pathogenic in HS as described previously. This approach can be applied for therapeutic intervention, screening and prototyping.

## Data Availability

The raw data supporting the conclusions of this article will be made available by the authors, without undue reservation.
